# A novel functional polymorphism of *GFAP* decrease glioblastoma susceptibility through inhibiting the binding of miR-139

**DOI:** 10.18632/aging.101442

**Published:** 2018-05-10

**Authors:** Jie Wang, Ming-Lei Wang, Chang-Hui Wang, Shu-Yan Sun, Han-Bing Zhang, Yang-Yang Jiang, Qi-Wu Xu, Ying Wang, Shi-Xin Gu

**Affiliations:** 1 Department of Neurosurgery, The Shanghai Neuromedical Center, Qingdao University, Shanghai, China; 2 Department of Neurosurgery, PuTuo District People's Hospital, Shanghai, China; 3 Department of Neurosurgery, Huashan Hospital, Fudan University, Shanghai, China; 4 Department of pathology, People's Hospital of Rizhao, Rizhao, Shandong, China; 5 Department of Cardiovascular Surgery of the First Affiliated Hospital& Institute for Cardiovascular Science, Soochow University, Suzhou, Jiangsu, China; * Equal contribution

**Keywords:** GFAP, functional polymorphism, glioblastoma, miR-139, chemoresistance, metastasis

## Abstract

Glioblastoma (GBM) is the most commonly diagnosed solid tumor outside the central nervous system. However, genetic factors underlying GBM remain largely unclear. Previous studies indicated that Glial fibrillary acidic protein (*GFAP*) might play an important role in the aggressiveness of GBM and also contributed to its poor overall survival. The present study aims to test (1) the associations between *GFAP* single nucleotide polymorphisms (SNPs) and GBM cells chemoresistance and metastasis, and (2) the molecular mechanism accounting for their effects. Four tagging SNPs of *GFAP* were initially genotyped in 667 subjects and the significant SNP was further analyzed via online bioinformatical tools. SNP rs11558961 was found to be significantly associated with GBM susceptibility. It was predicted to influence microRNA(miR)-139 binding to 3'UTR of *GFAP* gene. In functional experiments, we found that cells transfected with rs11558961 G-allele constructs had lower baseline luciferase activities and were more responsive to miR-139 changes, compared to C-allele constructs. Moreover, rs11558961 C>G variant reduced the chemoresistance of GBM cells and migration capability. In conclusion, rs11558961 might influence the chemoresistance and progression of GBM cells via promoting the binding of miR-139, ultimately decrease the susceptibility of GBM. This investigation will shed light on the optimizing for clinical trial design and individualizing of therapeutic plans.

## Introduction

Glioblastoma (GBM) is the most fatal brain tumor and only a few prognostic factors such as age, initial Karnofsky performance status are known [1]. After diagnosis, the median survival of GBM is approximately 14 months [2] and GBM exhibits a high chemoresistance [3]. Therefore, identification and characterization of the role of genetic variations in predicting GBM risk are very important. It may help optimize clinical trial design and individualize therapeutic plans.

*GFAP* is very important in malignancy progression of brain neoplasms. It acts as an essential component of cytoskeleton and expresses almost exclusively in astrocytes [4]. Increasing evidences showed that *GFAP* level elevated in high-grade brain tumors, implicating that *GFAP* might be involved in the aggressiveness of brain tumors [5-7]. The polymorphism of *GFAP* gene was confirmed involving in neural dysfunction disease. C/C genotype at rs2070935 of the *GFAP* gene in Alexander disease was associated with an earlier onset and a more rapid progression of ambulatory disability compared with other genotypes [8]. According to candidate gene strategy, we chose 4 SNP located in *GFAP* gene and detected the association between SNPs and GBM risk.

miRNAs are small non-coding RNAs which participate in cell biological processes and always function as pivotal gene transcriptional regulators. They modulate gene expression via promoting mRNA degradation after binding to the 3'UTR [9]. With screening for functional SNPs associated with GBM risk, we found a significant SNP-rs11558961 locating in a miR-139 binding site. The variant affected *GFAP* expression with the presence of miR-139. Emerging evidences indicated that miR-139 was considered as a tumor suppressor in GBM, which played a role in repressing migration and malignantly proliferation of cancer cells [10,11]. This was in concordance with our findings, rs11558961 C>G variant resulted in a emerging miR-139 bind site and thus a reduced GBM risk. Taken together, to demonstrate the clear molecular mechanism between functional SNPs, miR-139, 3' UTR of *GFAP* mRNA, the malignant progression of cancer cells and the susceptibility for GBM is essential in the development of novel therapeutic strategies to suppress GBM progression.

In summary, we reported a functional SNP in GBM-rs11558961 in 3' UTR of *GFAP*. It significantly decreased GBM susceptibility and affected the binding of miR-139 to *GFAP* mRNA. This might be the potential mechanism of rs11558961 influencing the GBM susceptibility.

## RESULTS

### Association of SNPs with GBM susceptibility

rs11558961, rs1042329, rs8067254, and rs17027 were consistent with HWE in healthy controls, with the *P* value of 0.620, 0.078, 0.080 and 0.261 respectively. Among the 4 candidate SNPs, rs11558961 variant was significantly associated with a decreased susceptibility of GBM. Compared with wild type CC, CG and CG+GG genotypes were significantly associated with reduced risk of GBM (CG vs. CC: adjusted OR=0.68, 95%CI=0.49-0.95, *P*=0.022; CG+GG vs. CC: adjusted OR=0.69, 95%CI=0.50-0.94, *P*=0.020). In allele comparing model, the rs11558961-G allele was significantly associated with a decreased susceptibility of GBM in comparison to C allele (adjusted OR=0.77, 95%CI=0.61-0.97, *P*=0.042) (Table 1).

**Table 1:** Logistic regression analysis of associations between the genotypes of *GFAP* and GBM risk.

Variants	Genotypes	Cases No. (%) n=261	Controls No. (%) n=406	Crude OR (95%CI)	*P*^a^	Adjusted OR (95%CI)	*P*^b^
rs11558961	CC	138 (52.9)	178 (43.8)	1.00 (Reference)		1.00 (Reference)	
(HWE=0.620)	CG	99 (37.9)	185 (45.6)	0.69 (0.50-0.96)	**0.028**	0.68 (0.49-0.95)	**0.022**
	GG	24 (9.2)	43 (10.6)	0.89 (0.42-2.71)	1.000	0.86 (0.40-2.69)	1.000
	CG+GG	123 (47.1)	228 (56.2)	0.70 (0.51-0.95)	**0.023**	0.69 (0.50-0.94)	**0.020**
	C allele	375 (71.8)	541 (66.6)	1.00 (Reference)		1.00 (Reference)	
	G allele	147 (28.2)	271 (33.4)	0.78 (0.62-0.99)	**0.045**	0.77 (0.61-0.97)	**0.042**
rs1042329	GG	182 (69.7)	286(70.4)	1.00 (Reference)		1.00 (Reference)	
(HWE=0.078)	GA	79 (30.3)	115 (28.3)	1.09 (0.53-2.52)	0.395	1.08 (0.52-2.51)	0.439
	AA	0 (0)	5 (1.2)	0.89 (0.42-1.97)	0.459	0.88 (0.41-1.96)	0.517
	GA+AA	79 (30.3)	120 (29.5)	1.07 (0.51-2.43)	0.496	1.05 (0.49-2.41)	0.562
	G allele	443 (84.9)	687 (84.6)	1.00 (Reference)		1.00 (Reference)	
	A allele	79 (15.1)	125 (15.4)	0.99 (0.68-2.36)	1.000	0.99 (0.68-2.37)	1.000
rs8067254	GG	224 (85.8)	341 (84.0)	1.00 (Reference)		1.00 (Reference)	
(HWE=0.080)	GA	37 (14.2)	65 (16.0)	0.87 (0.56-1.34)	0.521	0.86 (0.54-1.32)	0.569
	AA	0 (0)	0 (0)	1.52 (0.03-76.94)	0.834	1.59 (0.03-77.01)	0.897
	GA+AA	37 (14.2)	65 (16.0)	0.87 (0.56-1.34)	0.521	0.86 (0.54-1.32)	0.569
	G allele	485 (92.9)	747 (92.0)	1.00 (Reference)		1.00 (Reference)	
	A allele	37 (7.1)	65 (8.0)	0.88 (0.58-1.33)	0.539	0.87 (0.57-1.31)	0.640
rs17027	AA	161 (61.7)	256 (63.1)	1.00 (Reference)		1.00 (Reference)	
(HWE=0.261)	AG	82 (31.4)	128 (31.5)	1.02 (0.72-1.43)	0.915	1.03 (0.72-1.45)	0.937
	GG	18 (6.9)	22 (5.4)	1.30 (0.68-2.50)	0.430	1.33 (0.69-2.51)	0.476
	AG+GG	100 (38.3)	150 (36.9)	1.06 (0.77-1.46)	0.721	1.07 (0.77-1.48)	0.789
	A allele	404 (77.4)	640 (78.8)	1.00 (Reference)		1.00 (Reference)	
	G allele	118 (22.6)	172 (21.2)	1.09 (0.83-1.42)	0.539	1.09 (0.83-1.43)	0.556

CI, confidence interval; OR, odds ratio.

^a^ Chi-square test for genotype distributions between cases and controls. ^b^ Adjusted for age, sex, smoking and drinking status in logistic regress models.

### Association of SNP rs11558961 Genotypes with miR-139 binding and *GFAP* expression

**Figure 1:**
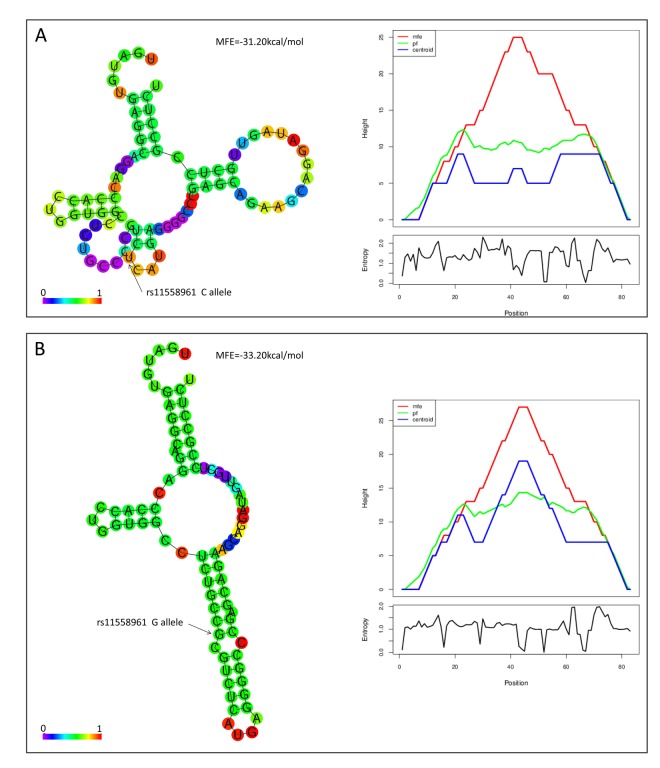
**rs11558961 affected secondary structure of *GFAP* mRNA. In silico prediction of rs11558961 impact on RNA folding structures.** The structures and Mountain plots for free energy or entropy corresponding to rs11558961C (**A**) or G allele (**B**). With G allele, there was an emerging miR-139 binding site exposed.

**Figure 2:**
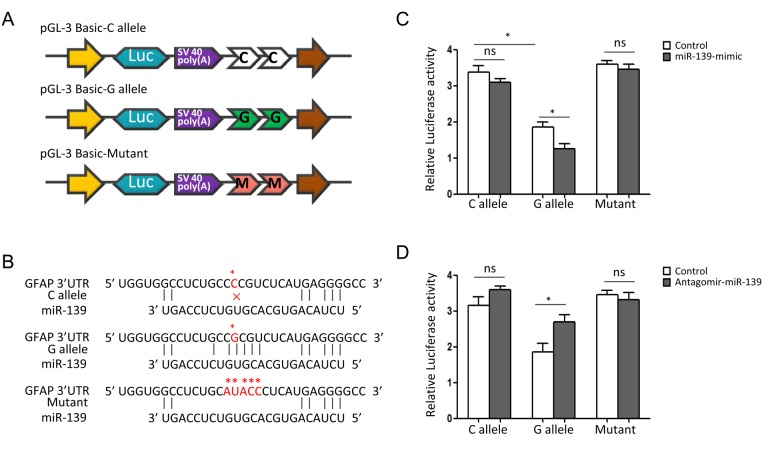
**SNP rs11558961 affects luciferase activities in U251 cells.** (**A**) The schematic diagrams of reporter plasmids construct. It shows the luciferase reporters carrying two copies of rs11558961-C allele, G allele or mutant sequence at the 3'UTR of luciferase gene. (**B**) This plot represents the theoretical miRNA-mRNA duplex pairing between miR-139 and *GFAP* 3′UTR with C, G allele or mutant. The C, G alleles and mutant are highlighted with asterisks (*). (**C**, **D**) Relative luciferase activities (vs. Renila luciferase) were measured in U251 cells transfected with C allele, G allele or mutant construct. Cells in different groups were treated with control miR, miR-139 mimic or antagomir-miR-139. Six replicates for each group and the experiment were repeated three times. *P<0.05, ns: non-significant compared between treatment group and control group.

Bioinformatical analyses suggested that rs11558961 was located at miR-139 binding site, the 3'UTR of *GFAP*. rs11558961 C>G variant affected secondary structure and minimum free energy of the 3'UTR mRNA (Fig. 1A, B). To evaluate the different affinity of C and G allele to miR-139, three luciferase reporter constructs were created (Fig. 2A). The G allele was predicted to bind with miR-139 but C allele not. The binding site in mutant sequence was completely destroyed (Fig. 2B). In GBM cells transfected with rs11558961-G allele construct, miR-139 mimic attenuated the luciferase activity by 30.63% while compared with control group; however, miR-139 mimic only reduced the luciferase activity by 7.58% in rs11558961-C allele group (Fig. 2C). Antagomir-miR-139 increased the luciferase activity by 42.31% in G allele group than that by 9.86% in C allele group (Fig. 2D). Mutant construct lost the miR-139 binding site did not present any obvious change. Based on the above evidences, miR-139 mimic could interfere *GFAP* expression in both rs11558961-C and G allele groups. Antagomir-miR-139 increased *GFAP* expression in C and G allele groups. Under the three conditions (control, miR-139 mimic, antagomir-miR-139), cells carrying G allele construct showed lower luciferase activity than those with C allele. Destruction of the miR-139 binding site prevented the effects of miR-139, antagomir-miR-139 on *GFAP* expression.

**Figure 3:**
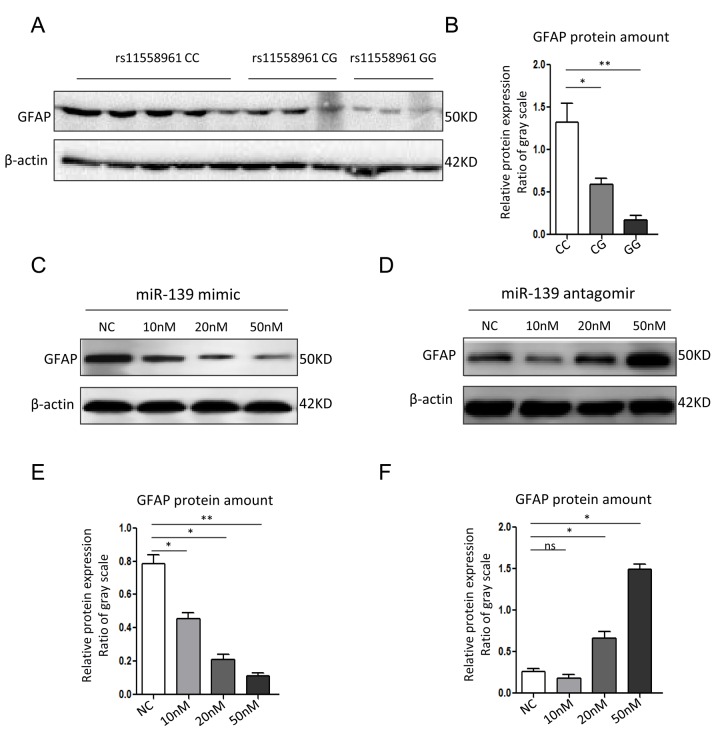
**miR-139 inhibited *GFAP* expression in U251 cells.** (**A**, **B**) Western blot analysis for *GFAP* expression in GBM tissues from patients with different genotypes (**A**) and corresponding quantitative analysis (**B**). (**C**, **D**) Western blot assay of *GFAP* in U251 cells transfected with miR-139 mimic or antagomir-miR-139 in different doses. (**E**, **F**) Gray scale quantification of the *GFAP* protein expression levels. Each experiment was repeated three times. *P<0.05, **P<0.01, ns: non-significant compared between treatment group and control group.

We further compared *GFAP* expression among GBM cells with different genotypes. The Western Blot data revealed that the expression level of GFAP were highest in CC group, and then in CG or GG group (Fig. 3A-B). In U251 cells transfected with miR-139 at different dose, *GFAP* expression was reduced dose-dependently by miR-139 mimic, and increased dose-dependently by antagomir-miR-139 (Fig. 3C-F).

### rs11558961 C>G variant reduced chemoresistance and increased imatinib-induced apoptosis

**Figure 4:**
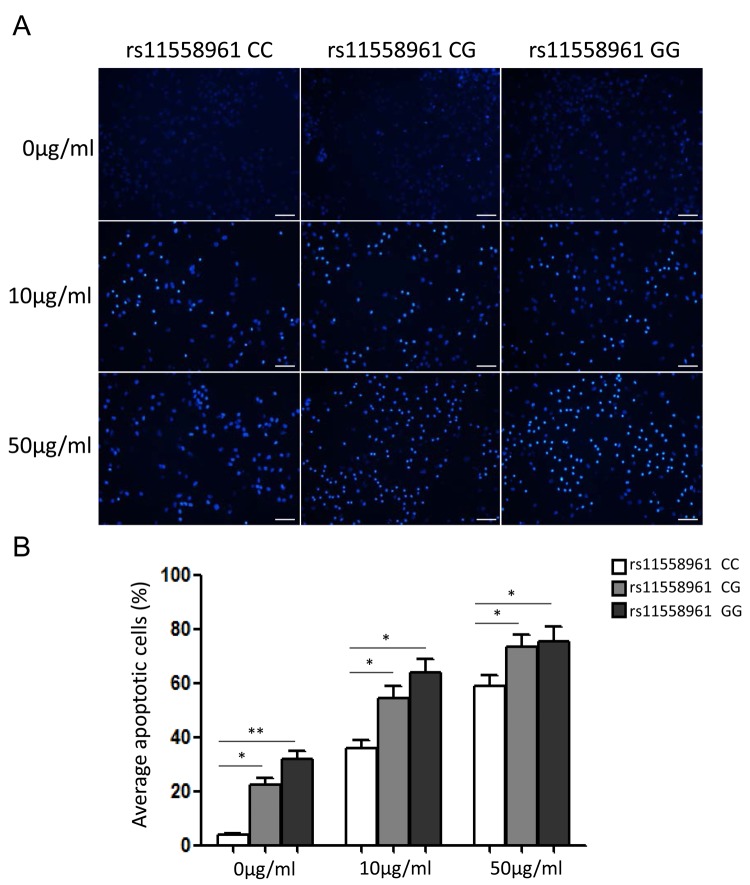
**rs11558961 C>G variant reduced chemoresistance and increased imatinib-induced apoptosis of GBM primary cells.** (**A**) The apoptosis status of cells carrying different genotypes were examined under a fluorescence microscope after Hoechst 33258 staining. (**B**) Quantification analysis by Image J software. All experiments were performed in triplicate. Data was presented as mean ±SD. Scale Bar = 100μm. *P<0.05, **P<0.01.

To evaluate the biological effect of rs11558961 on malignant phenotype of GBM cells in vivo, we measured the chemoresistance in GBM cells from patients with CC, CG and GG genotypes. As shown in Fig. 4, the chemoresistance was notably different among patients with rs11558961 CC, CG and GG genotypes. With the treatment of imatinib (10μg/ml, 50μg/ml), the average apoptotic cell ratio was lowest in CC group, followed by CG and then highest in GG group (10μg/ml: 36.21±6.03, 53.19±10.32 and 62.51±11.34; 50μg/ml: 59.37±7.98, 72.59±11.57 and 75.42±12.53 respectively). These results suggested that rs11558961 variant, which was related with *GFAP* expression, indeed increased imatinib-induced apoptosis, and reduced chemoresistance.

### rs11558961 variant suppressed the migration of GBM cells

**Figure 5:**
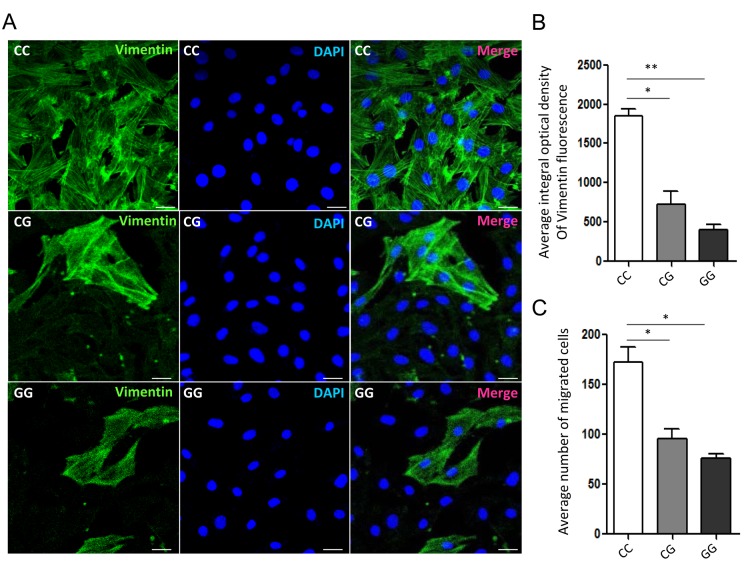
**Vimentin organization and migration of GBM primary cells with different genotypes of rs11558961.** (**A**) Representative confocal-microscopy graphs of immunostaining for Vimentin in rs11558961 CC, CG and GG cells. Green: Vimentin; Blue: DAPI. (**B**) Quantitative analysis of vimentin fluorescence in different groups. (**C**) Migration of cells with different genotypes. The experiment was repeated three times. Data were presented as mean ± SD. Scale bar = 50μm. *P<0.05, **P<0.01.

It has been proven that *GFAP* is a pro-tumorigenic factor in brain tumor [5-7]. To confirm the association between rs11558961 and malignant progression of GBM, we detected the vimentin expression and migration of GBM cells with CC, CG and GG genotypes. After stained with Alexa flour 488 conjugated antibody, we found that the vimentin expression level in CC groups was significantly higher than that in CG or GG group (average integral optical density: 1869.05±57.32, 692.31±82.11, 387.62±42.76, respectively). Vimentin is a primordial component of class-III intermediate filaments. It is involved in increased cell motility and migration capability of GBM, which is a hint of malignant progression [12]. Then we further examined the migration ability of cells in different groups. The data clarified that migration cells trans filtration membranes were the most in CC group, then CG and GG group (172.39±18.75, 92.51±12.18 and 75.29±10.21, respectively). Taken together, rs11558961 C>G variant substantially reduced vimentin expression and suppressed the migration of GBM cells (Fig. 5).

## DISCUSSION

**Figure 6:**
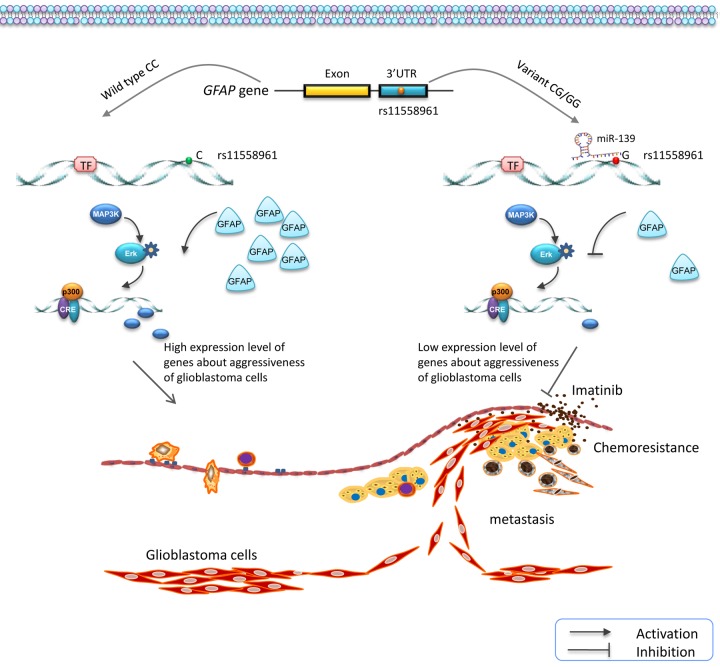
**Schematic diagram for the potential functions of rs11558961 in *GFAP* expression and progression of GBM.** rs11558961 variant G allele might decrease *GFAP* expression via forming a miR-139 binding site, and further suppress the metastasis and chemoresistance of GBM cells.

In the present study, we investigated the genetic effects of SNPs in *GFAP* gene on the progression of GBM. Four candidate SNPs were genotyped using MALDI-TOF MS in 261 patients and 406 healthy controls. A significant association between rs11558961 and decreased GBM susceptibility was identified. Variant G allele of rs11558961 reduced *GFAP* expression via interfering the binding of miR-139 and 3'UTR, resulting in low *GFAP* expression level, and further suppressing the chemoresistance and metastasis of GBM cells (Fig. 6).

rs11558961 was located at the 3'UTR of *GFAP* and predicted in a miR-139 binding site, with proximal transcription regulatory potential. While allele C changes to G, the secondary structure and minimum free energy of mRNA changed (Fig. 1); there was an emerging miR-139 binding site exposed. These analysis data were acquired using bioinformatical software-rSNP and SNPinfo. It confirmed the significance of this functional SNP in transcriptional regulation for *GFAP* gene. According to the results shown in Fig. 2-3, rs11558961 C>G change decreased the transcriptional activity, promoted miR-139 binding, therefore inhibited *GFAP* expression. To the best of our knowledge, the functional polymorphism-rs11558961 has not been reported before. This is the first time to report the potential function of rs11558961 in *GFAP* expression and GBM susceptibility.

Another interesting finding was that rs11558961 might be associated with chemoresistance, via fine tuning the *GFAP* expression. *GFAP* is important in malignant progression of brain neoplasm. It acts as an essential component of cytoskeleton and expresses almost exclusively in astrocytes [4]. Increasing evidences showed that *GFAP* level was elevated in high-grade brain tumors, implicating that *GFAP* might be involved in the aggressiveness of brain tumors [5-7]. Intermediate filament proteins promote cytoskeleton assembly/stabilization, and correlate with increased chemoresistance of cytotoxic agents [13]. Our study showed that rs11558961 C>G variant reduced the chemoresistance of GBM cells against imatinib. However, rs11558961 variant was proven associated with *GFAP* expression (Figs. 2-3). Therefore, rs11558961 might decrease the chemoresistance via suppressing *GFAP* expression. The subject carrying C allele is more prone to high-grade GBM because the chemoresistance of their cells tend to be higher than those with G allele.

Dysfunction of *GFAP* has been implicated in the astrocytes abnormality, due to the defect of cytokinesis and cell signal transduction. For example, heterozygous missense mutations of *GFAP* gene resulted in Alexander disease, a neurodegenerative disorder that affects children typically [14]. Generous studies revealed that epigenetic activation of *GFAP* or high *GFAP* expression was correlated with aggressiveness of brain tumor [5-7]. These reports suggested that *GFAP* was very important in nervous system disease. Vimentin expression and migration also played a important role in brain tumor cell survival and development. Thus, we evaluated the relationship between rs11558961, vimentin expression and migration of GBM cells. In immunofluorescence assay, the vimentin expression level in wild type group was significantly higher than that in variant group. In concordance with this result, the migration capability of GBM cells was higher in wild type group than that in variant groups. Vimentin is a primordial component of class-III intermediate filaments. It is involved in increased cell motility and migration capability of GBM, which is a hint of malignant progression [12]. Taken together, rs11558961 C>G variant substantially reduced vimentin expression and suppressed the migration of GBM cells, which was critical in progression of GBM cells.

There were several limitations in this study: (1) Since all the participants were almost collected from eastern Han Chinese population, the association between *GFAP* SNP and GBM risk might not be generalized to overall ethnic groups. (2) The large cohort, multi-center studies about relationship between rs11558961 and GBM susceptibility should be conducted to generate more potent statistical powers. (3) In functional experiments, the sample size is very small. Only 11 primary GBM samples limits the extrapolation of the study in human samples. Thus, more tissues should be collected to confirm the results. (4) GBM is a complicated disease. The genetic variation and the environmental effects of GBM contribute to the tumor heterogeneity and development. The potential molecular mechanism of *GFAP* promoting the aggressiveness of GBM is also important. Liu et al. revealed that retinoic acid (RA) and its derivatives, initiate RA-linked signaling transductions and induce chemotherapy sensitivity in GBM [15]. Moreover, changes of cellular RA balance in GBM might also be related to changes of *GFAP* expression and phenotypes. Therefore, it is necessary to clarify underlying mechanisms between the important intermediate filament-*GFAP* in GBM, cytoskeleton assembly and RA signaling responses. The further investigations should be performed.

In summary, we verified that rs11558961 variant in *GFAP* 3'UTR affected the miR-139 binding and *GFAP* expression in GBM for the first time. The G allele of rs11558961 predisposed hosts to downregulated *GFAP* expression and reduced GBM susceptibility. rs11558961variant genotype was a significant genetic protect factor. These findings will be meaningful in precaution, diagnosis and surveillance of GBM, as well as in the advancement of targeted therapy for this brain malignancy.

## MATERIALS AND METHODS

### Study population and postoperative follow-up

Consecutive GBM patients who received curative tumorectomy at Department of Neurosurgery in Zhenjiang first people's hospital, Changzhou first people's hospital, and the first affiliated hospital of Soochow University from Sep 2007 to Dec 2014. Healthy controls were randomly selected from people who received physical examination at Advanced Physical Examination Center. The demographics and clinical features of the GBM patients and controls involved in the study were listed in Table 2. Cases and controls were frequency-matched on gender and healthy controls were older than patients. All the patients were pathologically confirmed and signed the informed consent. Follow-up was performed via in person interview or telephone calls according to the standard epidemiologic procedure twice a year. The final date of follow-up was May 26, 2016. All the protocols conformed to the 1975 Declaration of Helsinki and were approved by the ethics committee.

**Table 2:** Demographic and clinical features of the study subjects.

**Characteristic**	**Case-control study**			
	**GBM cases (%)** **N=261**	**Controls (%)** **N=406**	***P***	
**Age (years)**				
Average (Range)	56.3 (22-81)	61.2 (27-92)	**<0.001**	
≤ 50	76 (29.1)	109 (26.8)	0.522	
>50	185 (70,.9)	297 (73.2)	**-**	
**Gender**				
Male	139 (53.3)	229 (56.4)	0.425	
Female	122 (46.7)	177 (43.6)	-	
**AJCC stage**				
I	167 (64.0)	-	-	
II	78 (29.9)	-	-	
III	16 (6.1)	-	-	
IV	0 (0)	-	-	

Abbreviation: AJCC: American Joint Committee on Cancer.

### Blood samples collection and GBM cells primary culture

Ethylene diamine tetraacetic acid (EDTA) anticoagulated peripheral blood samples were collected from patients before surgery and healthy controls. Genomic DNA was extracted using a RelaxGene Blood DNA System (TIANGEN biotech, Beijing, China) according to the manufacturer's instruction. The fresh surgical specimens of GBM from 11 patients were harvested and immediately immersed in ice-cold PBS containing 1% penicillin/streptomycin and 0.5% glutamine (Beyotime, Shanghai, China). Primary cell culture was performed within 60 min after surgery. As a variation of phenotype might occur at higher passages, we chose the cell cultures at the fourth passage for functional experiments.

### SNP selecting and genotyping

We selected 4 SNPs by analyzing *GFAP*-related Han Chinese data from 1000 Genome Project resources (http://www.1000genomes.org). The candidate SNPs should meet these criteria: (1) the minor allele frequency (MAF) > 0.05; (2) r^2^ < 0.80; (3) at the 3'UTR of *GFAP* gene. The significant SNP rs11558961 is located at a miR-139 binding site, with predicted proximal transcriptional regulatory potential (http://rsnp.psych.ac.cn/) (http://snpinfo.niehs.nih.gov/cgi-bin/snpinfo/snpfunc.cgi). rs1042329, rs8067254, and rs17027 were also predicted as functional SNPs, which were seated nearby other miRNA binding sites.

All SNPs of *GFAP* gene were genotyped using matrix-assisted laser desorption ionization time-of-flight mass spectrometry (MALDI-TOF MS). Amplification and single-base extension primers applied in multiple PCR were synthesized by Benegene (Benegene Biotechnology, Shanghai, China) (Table 3). The product of each sample was dispensed onto a 384-format SpectroCHIP with the MassARRAY Nanodispenser RS 1000. Then the MALDI-TOF MS assay was performed on a MassARRAY Compact Analyzer. Genotype calling was conducted using MassARRAY RT software version 3.0. Data was analyzed by MassARRAY Typer software 4.0.3 (Sequenom, San Diego, CA, USA). Genotyping quality was detected using Sanger sequencing of ~10% randomly selected samples, yielding a 100% concordance.

**Table 3:** Primers for SNP genotyping in PCR and single-base extension reaction of MALDI-TOF MS assay.

Gene(*GFAP*) Loci	Chr:position	Forward primer^a^	Reverse primer^a^	Extension primer	Amplicon size(bp)
rs11558961	17:44907319	linker-TTGCACTGTGCACGTTC	linker- TGGGGAAATGTGCCAG	GGATGTGATGTGA	165
rs1042329	17:44906346	linker-TGCCCCGTGCAGACTGGA	linker- TCCCACAATCCAGAGG	CGTGC AGACTGGA	119
rs8067254	17:44907009	linker- CAGTTCCCAGATAC	linker- GCACCTACTACATC	CTCCATCTCTGGCA	92
rs17027	17:44905825	linker- CTTCTTCGGCCTTAG	linker- GCTTTGACTGAGCAGAC	CCTTAGAGGGGAGA	106

^a^linker=10-mer linker ACGTTGGATG placed at 5'-end of each primer.

### Bioinformatic prediction of candidate SNPs

The SNP-flanking region RNA was online analyzed using SNPfold (http://ribosnitch.bio.unc.edu/Downloads/SNPfold/) and RNAfold (http://rna.tbi.univie.ac.at/cgi-bin/RNAWebSuite/RNAfold.cgi). SNPs in the 3'UTR were predicted for putative miRNA binding site applying three algorithms: miRBase (http://www.mirbase.org/search.shtml), PicTar (http://pictar.mdc- berlin.de/) and TargetScan (http://targetscan.org/).

### Quantitative real-time PCR analysis of miR-139

The expression level of miR-139 in U251 cells was detected using RNA-tailing and primer-extension real-time PCR according to the instructions of All-in-One miRNA Detection Kit (Genecopoeia, Guangzhou, China). miR-139 forward primer and miRNA universal adaptor PCR primer were provided by Genecopoeia corporation (Genecopoeia, Guangzhou, China).

### Luciferase reporter assay

To evaluate the binding of miR-139 to 3'UTR of *GFAP*, three reporter constructs carrying two copies of rs11558961-C allele, G allele or mutant sequences at the 3'UTR of luciferase gene were created. Firstly, three ~100 bp DNA sequences centered at rs11558961 (C or G allele) or mutant sequence were synthesized; then, two tandem copies of these sequences were cloned into pGL-3 Basic vector (Promega, Madison, WI, USA) using restriction enzyme sites- BamH I and Sal I. The schematic diagram for vector construction was shown in Fig. 2A. Day 1, U251 cells were seeded at 1×10^4^ cells/well in a 24-well plate. Day 2, cells were transfected with 0.8 μg pGL-3 Basic vectors (inserted with rs11558961- C allele, G allele or mutant sequences) and 0.16μg pRL-TK vector (Luciferase Assay System; Promega). Cells were transfected with mimic control, antagomir control, miR-139 mimic or antagomir miR-139 (GenePharma, Shanghai) in different group. Day 3, the luciferase activity was examined on Synergy H1 microplate reader (BioTek Instruments, Winooski, VT, USA) using Dual-luciferase Reporter Assay System (Promega, Madison, WI, USA) according to the instructions of manufactures. Results were represented as relative luciferase activity to pRL-TK.

### Imatinib-induced apoptosis

The GBM primary cells with rs11558961 CC (n=5), CG (n=3) or GG (n=3) genotypes (5×10^3^ per well) were incubated with imatinib at the concentration of 10μg/ml or 50μg/ml for 24 hours. The cells were then fixed with 4% paraform followed by staining with 200 μl Hoechst 33258 reagent (Beyotime, Shanghai, China) in the dark for 30 min. After being washed twice with PBS, cells were immediately photographed under an inversion fluorescence microscope (Olympus IX51, Tokyo, Japan) to determine ratios of apoptotic cells. In CC, CG or GG group, cells were quantified by counting at least 3 samples, 5 independent visual fields/sample, to determine the apoptotic ratio. Data was presented as mean ± SD.

### Cell migration assay

Cell migration measurements were performed using 24-well Transwell® units (8.0 μm pore, Costar Corning, NY, USA). In brief, different GBM primary cells (1×10^3^) in 100 μl were added to each matrigel-coated insert, and 500 μl DMEM supplemented with 10% FBS was added to the lower chamber. The plates were incubated at 37 °C, 5% CO2 for 24 h. The non-migrated cells in the insert were wiped away and the migrated cells were stained with ponceau. Finally, the images were captured with a phasecontrast microscope (Olympus IX51, Tokyo, Japan). There were 11 groups in this experiment, rs11558961 CC (n=5), CG (n=3) or GG (n=3). In each group, the migrated cells were quantified by counting at least 3 different samples, 5 independent visual fields/sample, to determine the average invasion ratio. Each assay was performed in triplicate.

### Statistical analysis

The differences of the demographic and clinical features, and frequencies of genotypes in case-control study were calculated by the Chi-square test (for categorical variables) or Student's t-test (for continuous variables). Hardy–Weinberg equilibrium (HWE) was detected by online analytical tools (https://ihg.gsf.de/cgi-bin/hw/hwa1.pl). The linkage disequilibrium (LD) of candidate SNPs was analyzed using HaploView v4.2 (Broad Institute, Cambridge, MA, USA). For the determination of main effect of SNPs, univariate and multivariate logistic regression models were conducted to generate odds ratio (ORs) and corresponding 95% confidence intervals (CIs) with adjustment for possible confounders, i.e. age, gender, drinking status and so on. All statistical tests were two-sided and performed using Statistical Program for Social Sciences (SPSS 16.0, Chicago, IL, USA) and R (http://www.r-project.org/). *P*<0.05 was considered as statistically significant.

## References

[1] Xiao Y, Decker PA, Rice T, McCoy LS, Smirnov I, Patoka JS, Hansen HM, Wiemels JL, Tihan T, Prados MD, Chang SM, Berger MS, Kosel ML, et al. SSBP2 variants are associated with survival in glioblastoma patients. Clin Cancer Res. 2012; 18:3154–62. 10.1158/1078-0432.CCR-11-2778 22472174 PMC3607457

[2] Das S, Marsden PA. Angiogenesis in glioblastoma. N Engl J Med. 2013; 369:1561–63. 10.1056/NEJMcibr1309402 24131182 PMC5378489

[3] Loftus JC, Dhruv H, Tuncali S, Kloss J, Yang Z, Schumacher CA, Cao B, Williams BO, Eschbacher JM, Ross JT, Tran NL. TROY (TNFRSF19) promotes glioblastoma survival signaling and therapeutic resistance. Mol Cancer Res. 2013; 11:865–74. 10.1158/1541-7786.MCR-13-0008 23699535 PMC3748253

[4] Eng LF, Ghirnikar RS, Lee YL. Glial fibrillary acidic protein: GFAP-thirty-one years (1969-2000). Neurochem Res. 2000; 25:1439–51. 10.1023/A:1007677003387 11059815

[5] Brommeland T, Rosengren L, Fridlund S, Hennig R, Isaksen V. Serum levels of glial fibrillary acidic protein correlate to tumour volume of high-grade gliomas. Acta Neurol Scand. 2007; 116:380–84. 10.1111/j.1600-0404.2007.00889.x 17986096

[6] Husain H, Savage W, Grossman SA, Ye X, Burger PC, Everett A, Bettegowda C, Diaz LA Jr, Blair C, Romans KE, Holdhoff M. Pre- and post-operative plasma glial fibrillary acidic protein levels in patients with newly diagnosed gliomas. J Neurooncol. 2012; 109:123–27. 10.1007/s11060-012-0874-8 22492246 PMC3715051

[7] Jung CS, Foerch C, Schänzer A, Heck A, Plate KH, Seifert V, Steinmetz H, Raabe A, Sitzer M. Serum GFAP is a diagnostic marker for glioblastoma multiforme. Brain. 2007; 130:3336–41. 10.1093/brain/awm263 17998256

[8] Yoshida T, Mizuta I, Saito K, Ohara R, Kurisaki H, Ohnari K, Riku Y, Hayashi Y, Suzuki H, Shii H, Fujiwara Y, Yonezu T, Nagaishi A, Nakagawa M. Effects of a polymorphism in the GFAP promoter on the age of onset and ambulatory disability in late-onset Alexander disease. J Hum Genet. 2013; 58:635–38. 10.1038/jhg.2013.83 23903069

[9] Ricci EP, Limousin T, Soto-Rifo R, Rubilar PS, Decimo D, Ohlmann T. miRNA repression of translation in vitro takes place during 43S ribosomal scanning. Nucleic Acids Res. 2013; 41:586–98. 10.1093/nar/gks1076 23161679 PMC3592420

[10] Yue S, Wang L, Zhang H, Min Y, Lou Y, Sun H, Jiang Y, Zhang W, Liang A, Guo Y, Chen P, Lv G, Wang L, et al. miR-139-5p suppresses cancer cell migration and invasion through targeting ZEB1 and ZEB2 in GBM. Tumour Biol. 2015; 36:6741–49. 10.1007/s13277-015-3372-8 25833697

[11] Chen Z, Yu T, Cabay RJ, Jin Y, Mahjabeen I, Luan X, Huang L, Dai Y, Zhou X. miR-486-3p, miR-139-5p, and miR-21 as Biomarkers for the Detection of Oral Tongue Squamous Cell Carcinoma. Biomark Cancer. 2017; 9:1–8. 10.1177/1179299X1700900001 28096697 PMC5224348

[12] Wang L, Zhang J, Banerjee S, Barnes L, Barnes L, Sajja V, Liu Y, Guo B, Du Y, Agarwal MK, Wald DN, Wang Q, Yang J. Sumoylation of vimentin354 is associated with PIAS3 inhibition of glioma cell migration. Oncotarget. 2010; 1:620–27. 10.18632/oncotarget.101101 21317457 PMC3248133

[13] Fortier AM, Asselin E, Cadrin M. Keratin 8 and 18 loss in epithelial cancer cells increases collective cell migration and cisplatin sensitivity through claudin1 up-regulation. J Biol Chem. 2013; 288:11555–71. 10.1074/jbc.M112.428920 23449973 PMC3630871

[14] Brenner M, Johnson AB, Boespflug-Tanguy O, Rodriguez D, Goldman JE, Messing A. Mutations in GFAP, encoding glial fibrillary acidic protein, are associated with Alexander disease. Nat Genet. 2001; 27:117–20. 10.1038/83679 11138011

[15] Liu RZ, Li S, Garcia E, Glubrecht DD, Poon HY, Easaw JC, Godbout R. Association between cytoplasmic CRABP2, altered retinoic acid signaling, and poor prognosis in glioblastoma. Glia. 2016; 64:963–76. 10.1002/glia.22976 26893190 PMC5595534

